# Evaluation of Roadside LiDAR-Based and Vision-Based Multi-Model All-Traffic Trajectory Data

**DOI:** 10.3390/s23125377

**Published:** 2023-06-06

**Authors:** Fei Guan, Hao Xu, Yuan Tian

**Affiliations:** 1 Department of Civil & Environmental Engineering, University of Nevada, 1664 N. Virginia St., Reno, NV 89557, USA; 2 School of Qilu Transportation, Shandong University, 17921 Jingshi Rd., Jinan 250014, China

**Keywords:** roadside LiDAR, roadway sensor, traffic trajectory data

## Abstract

Trajectory data has gained increasing attention in the transportation industry due to its capability of providing valuable spatiotemporal information. Recent advancements have introduced a new type of multi-model all-traffic trajectory data which provides high-frequency trajectories of various road users, including vehicles, pedestrians, and bicyclists. This data offers enhanced accuracy, higher frequency, and full detection penetration, making it ideal for microscopic traffic analysis. In this study, we compare and evaluate trajectory data collected from two prevalent roadside sensors: LiDAR and camera (computer vision). The comparison is conducted at the same intersection and over the same time period. Our findings reveal that current LiDAR-based trajectory data exhibits a broader detection range and is less affected by poor lighting conditions compared to computer vision-based data. Both sensors demonstrate acceptable performance for volume counting during daylight hours, but LiDAR-based data maintains more consistent accuracy at night, particularly in pedestrian counting. Furthermore, our analysis demonstrates that, after applying smoothing techniques, both LiDAR and computer vision systems accurately measure vehicle speeds, while vision-based data show greater fluctuations in pedestrian speed measurements. Overall, this study provides insights into the advantages and disadvantages of LiDAR-based and computer vision-based trajectory data, serving as a valuable reference for researchers, engineers, and other trajectory data users in selecting the most appropriate sensor for their specific needs.

## 1. Introduction

For decades, researchers have been dedicated to improving traffic operations and traffic safety by studying driving behaviors and interactions between road users. Field traffic data plays a critical role in these studies, and its quality heavily relies on the development of sensor technology. In the early days of traffic research, traffic data collection involved manual methods, such as human observation and stopwatches, which had limitations in terms of data richness and accuracy [1,2].

With the continued increase in traffic volume, promoting the utilization of existing transportation networks is deemed more cost-effective compared to constructing new roads. Consequently, there is a greater need for more accurate and higher-quality traffic data to enhance the efficiency of the transportation network. In order to obtain more precise data, researchers and engineers have devised various solutions. In the late 1920s, a horn-based sensor was introduced as the first solution, which was triggered by the sound of a vehicle’s horn. Around the same time, an on-roadway treadle sensor was invented. This sensor, comprised of two metal plates, could collect pass-presence information by detecting pressure variations [3]. Despite having some mechanical issues, this sensor remained a popular choice for detecting vehicles at actuated traffic signals for many years. Additionally, an electro-pneumatic sensor was developed to overcome these mechanical problems. However, due to its high cost and limited counting accuracy, this sensor was not widely adopted [1].

While treadle sensors demonstrated satisfactory performance in detecting vehicle presence, engineers were still unsatisfied due to their susceptibility to damage when placed on the road surface. These sensors could be easily lifted by snowplows, requiring reinstallation after road resurfacing. These challenges prompted researchers and engineers to explore traffic sensors that could be permanently installed in or over the road, addressing these limitations.

Inductive loop detectors, which are installed in the road to provide vehicle pass/presence information, quickly gained dominance in the market due to their good performance, flexibility of design, and durability. Although a single loop detector could only collect pass/presence information for a specific location within one lane, by combining multiple loop detectors, various traffic parameters such as speed, vehicle length, and density could be derived through algorithms. Nowadays, over many decades of use, engineers have developed a mature installation and maintenance standard for inductive loop detectors, making them one of the most widely used sensors worldwide.

Despite researchers and engineers finding inductive loop detectors advantageous, they never stopped seeking other alternative sensor options due to some inherent limitations of loop detectors that could not be overlooked. Firstly, the data obtained from the loop detector is not accurate enough to facilitate advanced microscopic traffic analysis because loop detectors can only measure the traffic flow passing fixed cross lines. Secondly, the data quality collected by loop detectors may be affected by the pavement conditions, weather, and any operations to the road that can affect loop integrity. Thirdly, although in-roadway sensors are not as easy-broken as on-roadway sensors, maintenance costs tend to be higher due to the potential need for road closures.

In addition to the inductive loop detector, various traffic sensors have been deployed over the past few decades, including infrared sensors, ultrasonic detectors, acoustic detectors, magnetometers, and more. They have all achieved a certain degree of success in field traffic data collection, but due to the high cost and lack of comprehensive standards, they are not widely used.

Since the 2000s, researchers have started to seek traffic data containing dynamic temporal-spatial information. This type of traffic data needs to be able to provide the location of road users at a high frequency in order to generate vehicle moving trajectories. There are three main advantages of this type of data. Firstly, using high-frequency trajectory data can generate a continuous smooth time-space diagram, which improves studying traffic flow and understanding microscopic driving behavior. Secondly, macroscopic trajectory data provides precise origin-destination information, which greatly helps city planners to better estimate the number of trips and link travel time, and track trip distribution. Thirdly, trajectory data is good for the detection of some unique events, such as pedestrian Jaywalking and vehicle red-light running. In addition, trajectory data can also be used to perform some initiative-taking traffic safety analyses, such as near-miss detection. These diverse benefits have made researchers increasingly pursue this type of data.

In the initial stages, to get trajectory data, researchers used footage from the traffic camera to track the vehicles by recording the location of vehicles frame-by-frame manually, which is time-consuming and prone to errors. More recently, with advancements in communication and sensing technology, a wide range of in-vehicle devices, such as global positioning systems (GPS) and cellular phones, have been employed to collect trajectory data [4]. Vehicles equipped with these devices, known as probe vehicles or floating vehicles, continuously update their location information through wireless communications while traveling on the road [5]. Although this trajectory data is valuable, it still faces certain limitations: Firstly, since only a portion of vehicles is equipped with these devices, the data collected cannot fully represent real-time traffic conditions [6]. Secondly, the data quality is influenced by various aspects, such as the loss of cellular phone signal and inaccurate GPS positioning, among other reasons. Third, most providers of such data primarily include vehicle data, while other road users, such as pedestrians and bicyclists, are not included.

Obtaining multi-model all-traffic trajectory data from onboard devices is not a suitable choice due to the fact that not all road users carry location-tracking and communicating devices. As a result, scientists have turned their attention back to the roadway sensor since the roadway sensor can theoretically detect all objects that are not obstructed within the detection range. Up to now, there are two popular solutions. One is the roadside LiDAR-based solution, which uses 360-degree roadside LiDAR. It establishes a local coordinate system centered on LiDAR to detect the location of the object by calculating the time difference between the time the laser beam emitted from the sensor and reflected back to the sensor from the object. The other solution is a vision-based approach, which has advanced with the development of information technology. Researchers can now automatically identify the vehicles and pedestrians in each frame of the camera video and extract the location information using computer deep learning technology.

While both LiDAR-based and vision-based methods are capable of generating multi-model all-traffic trajectory data, the principles of data generation and processing are different. The differences will be elaborated upon in the subsequent section. The primary objective of this paper is to compare the difference between these two methods, evaluating the data quality by comparing the data output of both methods during the same period and at the same intersection. This paper will provide a summary of the advantages and disadvantages of LiDAR-based and vision-based trajectory data, which could be a reference for agencies and researchers to use when considering which sensor to use.

This paper is structured as follows: Section 2 provides a literature review of diverse types of trajectory data sources, introduces current applications on them, and explains the working principle of LiDAR-based and vision-based trajectory data. Section 3 presents a comparison of the data quality of two sources in terms of detection range, counting accuracy in different lighting conditions for both vehicles and pedestrians, and speed detection. Section 4 provides a conclusion, summarizes the contribution and limitations, and also points out future works.

## 2. Literature Review

### 2.1. Different Types of Traffic Trajectory Data

#### 2.1.1. Vision-Based Trajectory Data

Cameras have been utilized in the transportation field for many years. Initially, they were used to taking photos and backup videos for traffic violation cases such as speeding. To track road users, researchers in the early years would record the changing location of the interested road users based on the frame-by-frame images from video cameras [7]. However, this method is time-consuming, and the data quality is prone to human errors which cannot be predicted and avoided.

**Figure 1:**
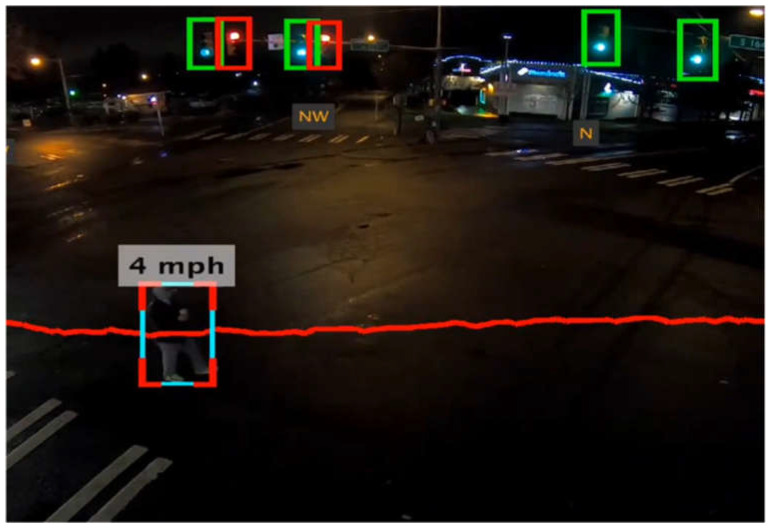
Pedestrian crossing captured by computer vision [10].

In modern times, with the advancement of computer vision technology, road users can be automatically identified and tracked in videos using algorithms [8]. Through machine learning algorithms, the background noise can be filtered out, traffic lanes can be identified, and all the moving objects can be recognized and categorized, as shown in Figure 1. Converting the moving objects in the video into trajectory data with spatial coordinates poses a significant challenge, which involves generating depth information from the video. The principles and detailed explanation are provided in Section 2.2. Nevertheless, the accuracy of this advanced vision-based trajectory data is highly dependent on the quality of the videos [9]. 

Both detection accuracy and range have been questioned in extreme weather, poor lighting, and low-resolution conditions [11]. 

#### 2.1.2. Radar-Based Trajectory Data

Radar has been applied in the transportation field for speed measurement and vehicle existence detection for decades due to its strong Doppler velocity sensitivity and resistance to adverse weather conditions [12,13]. Since Radar provides relative distance information between the detected target and the sensor, Radar is considered a potentially reliable source of trajectory data. However, Radar suffers from its limited angular resolution, causing confusion when multiple targets exist within its beamwidth [14]. This limitation hinders the guarantee of high-quality trajectory data. To address this issue, researchers have proposed Radar and camera fusion techniques to enhance target classification and tracking [15,16]. It shows good progression, but two main challenges persist. Firstly, performance in complex scenarios is often compromised, leading to missing objects and interference from background noise due to imperfect algorithms, target occlusion, or changes in the background. Secondly, the localization accuracy decreases as the distance between the target and the sensor increases [17].

Currently, with remarkable advancements in sensing technology, advanced Radars, such as Inverse Synthetic Aperture Radar (ISAR) [18,19], millimeter-wave linear frequency-modulated continuous-wave (LFMCW) radar [20], and 79 GHz ultra-bandwidth radar [21], have overcome the limitations of low resolution and demonstrated their viability for traffic surveillance.

#### 2.1.3. LiDAR-Based Trajectory Data

**Figure 2:**
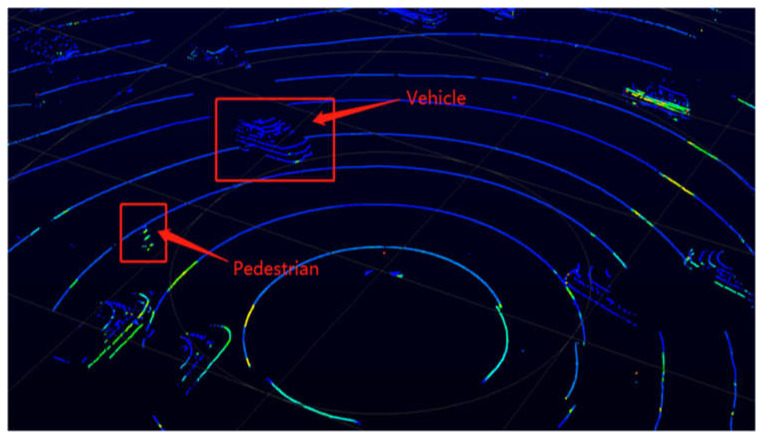
View of raw LiDAR data from Veloview.

Similar to Radar, LiDAR is a remote sensing technology for determining variable distance by measuring the time difference between the laser being transmitted from the transmitter and reflected back to the receiver. This technology finds extensive applications across various fields. In the transportation domain, LiDAR is predominantly employed as an onboard sensor for autonomous vehicles, owing to its favorable performance in low-light conditions and long detection range. However, there has been a growing interest in exploring the potential of LiDAR as a roadside sensor in recent years. Roadside LiDAR systems utilize distance data to perform three-dimensional (3D) stereo modeling, generating dynamic point cloud 3D models over time, as depicted in Figure 2. Through processes such as background filtering, clustering, and referencing, individual road users can be classified and tracked effectively [22].

#### 2.1.4. Other Types of Trajectory Data

Recall trip data: The prototype of trajectory data is the recall trip data. Recall trip data typically comprises basic trip details, such as the origin and destination locations, along with their corresponding timestamps. Additional information, such as intermediate stop-by locations and their timestamps, may also be included in more detailed datasets. In the early stages, this type of data was commonly collected through telephone interviews or daily travel diaries. However, it is important to acknowledge that driver-self-reported recall trip data may be subject to accuracy limitations. This is because of the fact that drivers often struggle to accurately recollect all the places they have visited and the exact times of arrival at each location. Consequently, shorter trips, such as visits to the pharmacy or post office, are more susceptible to being omitted. 

Global Positioning System (GPS) data: Initially limited to military use, GPS has been made available for civilian use since the 1980s. GPS originally appeared in the transportation field in the late 1990s to be an alternative to recall trip data. The Federal Highway Administration (FHWA) and the U.S. Department of Transportation (USDOT) deployed GPS devices in some private cars in the Lexington area to compare with recall data [23]. Presently, most smartphones and cars come equipped with built-in GPS modules, inspiring engineers to develop more GPS-based trajectory data collection methods as a replacement for dedicated GPS devices [24]. 

Smartphone-based trajectory data has gained popularity due to the utilization of mobile signals and internet connectivity to enhance GPS positioning accuracy. Additionally, this approach reduces costs and allows for the collection of data from non-vehicle road users, such as pedestrians and bicyclists [25]. However, research indicates that smartphones have a shorter warm-up time to provide the initial location but also have drawbacks such as shorter battery life and lower positioning accuracy compared to dedicated GPS devices [26].

Another emerging source of GPS-based trajectory is provided by some connected-vehicle (CV) original equipment manufacturers (OEMs). These connected vehicles are equipped with built-in GPS devices that continuously update real-time information to the OEMs’ data centers. As a result, OEMs can generate anonymized trajectory data, including geolocation, timestamp, heading, speed, and acceleration/deceleration information [27]. This type of data has gained popularity as it covers a wide range of cities and can be readily obtained from third-party commercial data providers. However, one limitation is that the penetration rate of connected vehicles remains relatively low, and the rates vary across different areas and roads, requiring further investigation and analysis.

### 2.2. Trajectory Data Processing

#### 2.2.1. LiDAR-Based Data Processing

Background filtering: Background filtering is a crucial initial step in the processing of LiDAR data. Its purpose is to effectively remove irrelevant data points that do not contribute to the research objectives, such as those reflected back from trees, buildings, and the ground. Meanwhile, it aims to retain the necessary data points representing objects of interest, such as cars, pedestrians, or wildlife. The challenge lies in accurately distinguishing between the points that should be preserved and those that should be filtered out within a dynamic point cloud output. Due to the slight LiDAR vibration and the influence of wind, the points reflected by objects are not entirely static, which adds complexity to the background filtering process. To address this challenge, a method named 3D density statistic filtering (3DDSF) has been used.

This method employs a process that involves overlapping the output of multiple LiDAR frames and dividing the space into small cubes with a side length of 0.05 mm. Each cube’s point count is recorded, and based on whether the number of points in a cube exceeds a predefined threshold, it is classified as either background or non-background. The threshold values are determined through machine learning, with different cubes having different thresholds. The distance between the cube and the LiDAR is a crucial factor in determining the threshold. Once all the cubes are defined, the points within the cubes classified as background are filtered out, retaining only the points within the cubes defined as “non-background”. This method achieves a remarkable accuracy of 99.2% [22].

The next step is object clustering. This step aims to classify each filtered point based on its similarity. To achieve this, an improved version of the density-based spatial clustering applications with noise (DBSCAN) method was employed, utilizing an adaptive minimum number of points (MinPts) value. This approach offers the advantage of not requiring a predefined number of objects (road users), which is particularly valuable in traffic analysis where the exact number of road users cannot be determined in advance. The method categorizes all points in the dataset into core points, border points, and noise while defining two important parameters: the minimum number of points (MinPts) and the search radius (ε). If the number of similar points within the search radius exceeds the minimum number of points (MinPts), these points are considered part of the same object. Different objects have a different minimum number of points (MinPts) values, with smaller values assigned to objects farther away from the LiDAR.

The last step in the data processing pipeline is object tracking, which involves obtaining the trajectory of the same object across different frames. The main challenge in this step is determining whether the point cloud corresponds to the same object in different frames. Our team has developed a prediction method based on Kalman filtering to address this challenge. By analyzing the position and speed of the objects, we can predict where they are likely to appear in the next frame and define a search area accordingly. Objects within this prediction range in the subsequent frame are considered to be the same object. If the search area in the second frame contains multiple objects, the one closest to the position of the object in the previous frame is selected as the associated object. Once the associated object is identified, its position and velocity measurements are updated and used to predict the object’s location in the next frame. If no suitable candidate is found within a certain number of frames within the prediction area of an object, the tracking process is terminated.

#### 2.2.2. Vision-Based Data Processing

The principle of generating vision-based trajectories differs from the LiDAR-based method. In the case of LiDAR, the raw data consists of point clouds captured over different frames, with each point’s relative spatial location easily determined based on its direction and distance information from the LiDAR origin. In contrast, the raw data in vision-based systems comprises video data, consisting of two-dimensional (2D) images that change over time. Each image consists of multiple pixels, which provide color information. The main challenge in this method lies in detecting objects and determining their spatial information based on 2D video data.

Stereo cameras have emerged as a popular solution to address this challenge. By utilizing multiple cameras, objects can be captured from different angles, allowing for the estimation of object depth through triangulation. The following section will outline the general procedure of how a stereo-camera tracking system operates.

The first step in the tracking system involves extracting road users from the video. For vision-based object extraction, there are two major approaches: motion-based and appearance-based. The motion-based method utilizes multiple frames and focuses on changes in image color. It involves filtering the background and extracting the color changes observed over different time frames. The limitation of this method is that this method can only capture changes in the image and may mistakenly classify stationary objects as part of the background, resulting in their removal. Additionally, this method may not remove noise effectively. The appearance-based method has gained popularity. It leverages artificial intelligence and machine learning techniques to directly identify objects based on their unique visual features, such as shape, color, and texture. This method is capable of detecting stationary objects and exhibits better resistance to noise. It also facilitates the subsequent object-tracking process, which will be discussed in detail in the object-tracking section. However, it should be noted that the appearance-based method significantly increases computational complexity.

**Figure 3:**
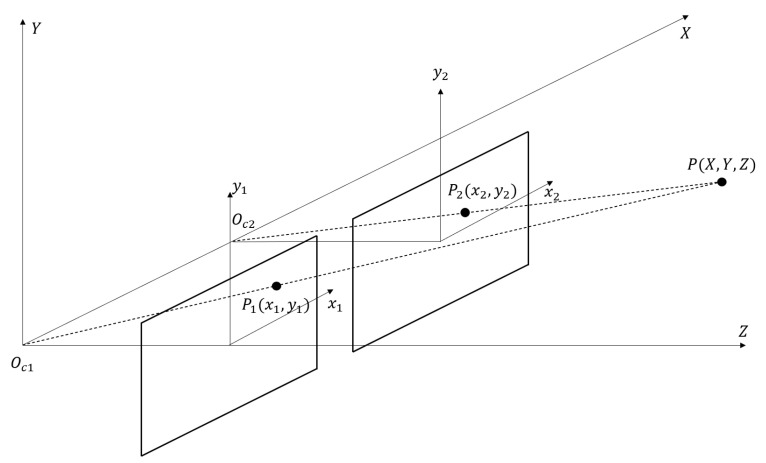
How stereo cameras generate depth information.

Once the same unique feature of an object is captured by two or more cameras, the depth of the object can be estimated using triangulation calculation. Depth is the distance between the image plane and the corresponding object, which is a critical parameter used to determine the spatial location of the object. Figure 3 illustrates the configuration of a simple stereo imaging system: a feature point P, which has a global three-dimensional coordinate of (X, Y, Z), is projected onto two parallel camera planes, and the projection has a local two-dimensional coordinate of (x_1_, y_1_) and (x_2_, y_2_), respectively. The local coordinates can be automatically obtained by a transformation matrix, which is determined by the specifications of the hardware (cameras). Once the relative distance between two cameras, i.e., the distance between two optical centers, O_c1_ and O_c2_ is obtained, the relationship between P, P1, and P2 can be addressed, then the global coordinate of P can be calculated based on their triangle relationship and the transformation matrix.

After detection and 3D mapping, the trajectory of the object, i.e., the location of the object in every time frame, can be generated by tracking the object. The challenge of object tracking is how to associate the same object in different frames. Similar to object detection, AI-feature-based technology can be used to recognize the same object in different frames by identifying the same unique appearance feature. Once the object is tracked, the trajectory points can be obtained based on the spatial information generated from depth estimation.

### 2.3. Applications on Trajectory Data

#### 2.3.1. Smart City

In general, trajectory data can be categorized into two types: macroscopic low-frequency trajectory data and microscopic high-frequency trajectory data. These two kinds of data serve different purposes and are sometimes used in combination. Macroscopic trajectory data typically includes long trajectory data of a subset of the road users in a road network, recording from the time when the driver starts the engine until the driver stops the engine. Due to limitations in communication technology and data size, the frequency of macro-trajectories is relatively low, with location information typically refreshed every few seconds. This kind of data is of great help to the planning of the large road network. Based on the trajectory of part of the total vehicles, the traffic flow and traffic volume of different road sections in the road network can be estimated [28,29,30]. The travel time, average speed, and route selection of vehicles passing through each road section provided by trajectory can be used to access the real-time traffic state of each road section [31,32]. The OD information provided by macro trajectory data can be used to analyze drivers’ travel patterns and investigate time-dependent attractive areas [33]. In addition, macroscopic trajectory data is also very helpful in traffic visualization. Since trajectory data has geolocation information, it can be readily projected onto the map for a series of visualization purposes, such as building an urban traffic congestion visualization system [34]. These visualizations can help better display the traffic states of the road network and help researchers provide strategies more effectively.

#### 2.3.2. Traffic Safety

Before the introduction of trajectory data, historical crash data was the major data source for traffic safety analysis. However, it had several limitations. Firstly, crashes may not be fully reported; those under-reporting crashes significantly influence the result of the analysis. Secondly, crashes occur randomly, and their frequency is relatively low. Collecting sufficient crash data to build meaningful statistical models requires a significant amount of time. To overcome these limitations, researchers have adopted a new approach to traffic safety analysis: studying traffic conflicts, also known as near misses or near-crashes [35]. Traffic conflicts refer to events on the road that have the potential to result in actual crashes. Unlike real crashes, traffic conflicts occur more frequently in real-world traffic, making them easier to capture and saving time on data collection. However, unlike crashes, traffic conflicts cannot be readily defined using a strict numerical equation. There are numerous parameters used to define traffic conflicts, such as Time to Accident (TA), Time-to-Collision (TTC), Post-encroachment Time (PET), and maximum deceleration (MaxD), among others. These parameters rely on the spatial-temporal relationships between road users provided by trajectory data. Researchers usually use various combinations of these parameters to set thresholds to define different kinds of traffic conflicts. By analyzing conflicts, researchers can gain better insights into drivers’ driving behavior [36], assess risk levels for roads and intersections, and predict possible risks and high-risk locations. A lane-changing risk index has been developed to evaluate the safety of lane-change events [37]. Another trajectory-based lane change study indicated that during lane changing, the following cars in the original lane and the target lane were the safest and riskiest vehicles, respectively [38]. In addition to the conflicts between vehicles, studies of pedestrian and bicycle safety were also conducted. Zhou et al. used vision-based trajectory data to investigate the probability of vehicle-pedestrian crashes [39]. Critical bus driving events extracted from GPS trajectory were utilized to be an alternative to surrogate safety measurement for bus stops [40]. Compared to the traditional historical crash data, the biggest advantage of conflict study based on trajectory data is that it can be used for proactive safety analysis [41], not only for reactive safety analysis. The causes of conflicts and crashes are considered to be the same [42], so by identifying and studying traffic conflicts, high-risk areas prone to crashes could be estimated [43]; in other words, the occurrence of crashes can be predicted [44,45,46,47,48]. Recent studies have applied trajectory-based conflict detection to real-time traffic infrastructures to warn drivers/pedestrians who are in risky situations [49].

#### 2.3.3. Environmental Impacts

Fuel consumption and greenhouse gas (GHG) emissions have become prominent issues of concern in recent years, prompting researchers to focus on finding ways to reduce vehicular traffic emissions. Vehicle exhaust emissions are a significant contributor to overall air pollution and greenhouse gas emissions, accounting for 45% of total air pollutants in the United States, according to the National Research Council (1995) [50]. Accurately estimating vehicle emissions during trips and understanding the factors that influence emission levels are crucial for effective gas mitigation strategies. However, estimating emissions accurately is challenging due to the variability in driving behavior and its impact on emissions [51]. The advent of trajectory data has significantly advanced research in this field. High-frequency trajectory data, providing instantaneous speed and acceleration information, serve as critical parameters for estimating vehicle fuel consumption and emissions [52]. 

There are lots of studies about the estimation of emission based on different types of trajectory data: Zhou et al. [53] proposed an estimation approach to combine a simplified MOVES model [54] with a mesoscopic simulation model. Kraschl-Hirschmann et al. [55] conducted a method using microscopic-simulated trajectory data. Martin et al. [56] applied a fuel consumption model to the NGSIM trajectory dataset to investigate the impact of traffic jams on fuel consumption and emissions. Chen et al. [57] estimated fuel consumption and emission based on reconstructed vehicle trajectories. There are also studies based on GPS data [58], floating car data [59], and mobile sensing data [60]. 

In terms of fuel optimization, Alsabaan et al. [61] proposed a method to optimize fuel cost and emission based on V2V communications. Wang et al. [62] developed a nonlinear model predictive control (MPC) method to reduce additional emissions due to human factors, i.e., inaccurate driver perception of space and/or time interval via longitudinal control of intelligent vehicles in a congested platoon. L.L. Lemos [63] conducted another intersection-level control to minimize vehicular air pollution.

In addition, Wu et al. [64] conducted a traffic experiment to get vehicle trajectories and each vehicle’s fuel rate by using 360-degree cameras and onboard diagnostic (OBD-II) scanners. This dataset contains accurate vehicle location information and fuel rate at each timestamp, which can be used to improve fuel consumption models and investigate engine emissions.

## 3. Comparison of Trajectory Output

**Figure 4:**
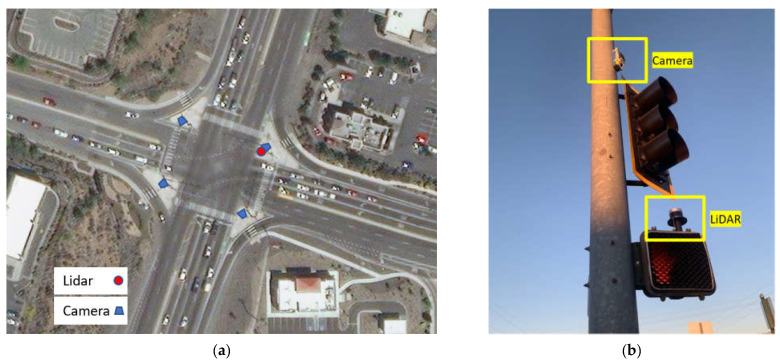
(**a**) Installation location of sensors; (**b**) field photo.

Regardless of what methods/technologies LiDAR and cameras use to process the raw data, their outputs are the same: the trajectories of road users with a certain frequency. This section compares the two trajectory outputs in terms of general location accuracy, volume counting accuracy, detection range, and speed. Both LiDAR-based and vision-based trajectory data in this study have a frequency of 10 Hz. The data collection took place at the intersection of Pyramid Way and Los Altos Pkwy in Sparks, NV, between 12:00 a.m. 23 December to 12:00 a.m. 24 December. Four GoPRO HERO8 cameras were placed at each corner to ensure coverage of all four legs of the intersection. A Velodyne VLP32 LiDAR sensor was installed at the northeast corner. The installation locations for the LiDAR sensor and cameras are depicted in Figure 4. It should be noted that because the separate right-turn lanes are not covered by the cameras, right-turn vehicles are not included in this study.

### 3.1. Trajectory General Location Accuracy

**Figure 5:**
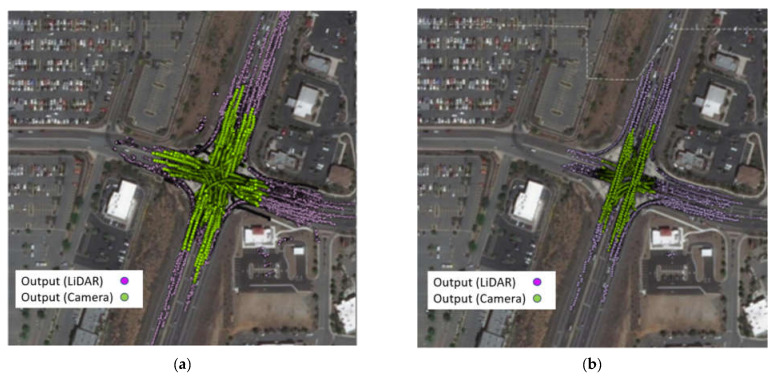
(**a**) Daytime trajectory overview; (**b**) nighttime trajectory overview.

Figure 5 displays two sets of 30 min data, one from 12:00 a.m. to 12:30 p.m. on 23 December (left) and from 4:00 a.m. to 4:30 a.m. on 24 December (right); the pink points represent the trajectory output from LiDAR, and the green points represent the trajectory output from the camera. Upon initial observation, it can be noted that both LiDAR and camera trajectories align well with the lanes. However, it is evident that the detection range of the vision-based trajectory is shorter compared to that of LiDAR, both in the daytime and nighttime conditions. A more detailed analysis of the detection range will be provided and discussed later in this article.

### 3.2. Vehicle Volume Count Accuracy

**Figure 6:**
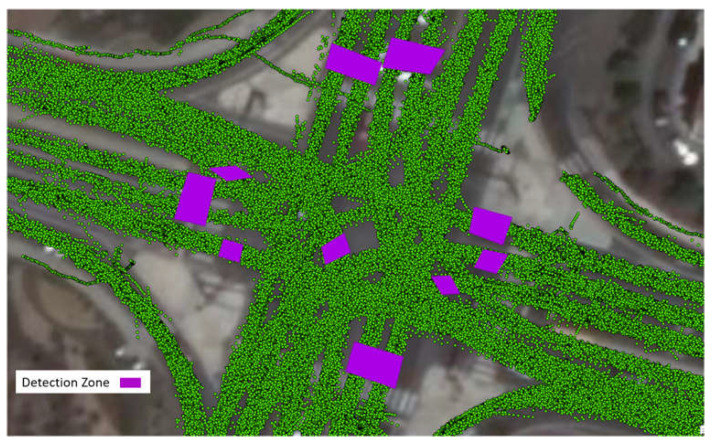
Detection zones for counting the volume.

The volume counts are not generated automatically but can be obtained by counting the different object IDs in each lane. To facilitate this process, detection zones are set up in the ArcGIS (ver. 10.0) [65], as illustrated in Figure 6. The number of unique objects within each detection zone can be automatically counted using the built-in function of ArcGIS, enabling the determination of the volume for each movement. The obtained volume counts are then compared with the ground truth volume data, which is manually collected by our team.

**Figure 7:**
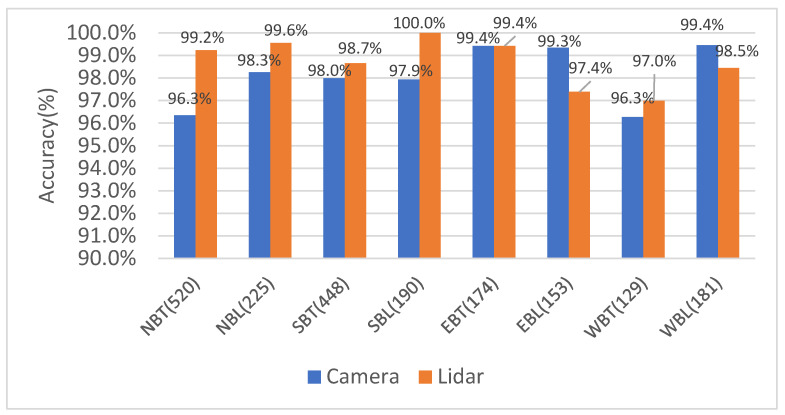
Volume count accuracy for daytime volume (24 December 2020, 12:00 p.m.–12:30 p.m.).

The results of volume count accuracy for each movement are presented in Figure 7. Each column represents the percentage of accuracy achieved by the sensor for the corresponding movement, including northbound through (NBT), northbound left turn (NBL), southbound through (SBT), southbound left turn (SBL), eastbound through (EBT), eastbound left turn (EBL), westbound through (WBT), and westbound left turn (WBL). The numbers within each bracket indicate the actual number of vehicles for each movement manually counted.

During the daytime peak time from 12:00 p.m. to 12:30 p.m. on 24 December, both the vision-based and LiDAR-based outputs demonstrated good performance, with an accuracy rate of over 96% for all movements. Overall, LiDAR achieved slightly better accuracy than computer vision, except for the eastbound left turn, northbound through, and northbound left turn movements, where cameras exhibited better accuracy.

For the nighttime period from 3:00 a.m. to 3:30 a.m. on 24 December, the accuracy of both LiDAR and cameras was evaluated by comparing the results with manually counted volume data. The LiDAR-based system achieved 100% accuracy for all movements. The camera-based system also performed well, reaching 100% accuracy for all movements except for the southbound through movement. For the southbound through movement, the camera’s accuracy rate was 81.5%, as the cameras were able to capture 22 out of the 27 vehicles.

### 3.3. Detection Range

The detection range of the two different sensors, LiDAR and cameras, was evaluated by setting four detection zones for each movement. Each zone was 50 ft long, with the front edge of the first zone aligned with the stop bar. The detection range was assessed by comparing the percentage difference of vehicles detected in each zone.

**Figure 8:**
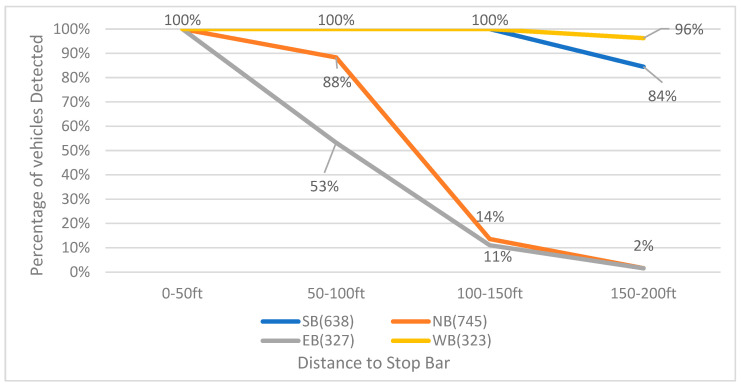
LiDAR-based vehicle detection rate for each bound (23 December 2020, 12:00 p.m.–12:30 p.m.).

Figure 8 illustrates the detection rate of LiDAR for different movements at varying distances from the stop bar. LiDAR achieves a 100% detection rate for vehicles within a distance of 0 to 50 ft from the stop bar. However, as the distance increases, the detection rates of the northbound and eastbound movements, which are farther from the installation position of LiDAR on the northeast side of the intersection, show a more significant decline. For the eastbound movement, the detection rate drops to 53% between 50 ft and 100 ft from the stop bar, further decreasing to 11% between 100 ft and 150 ft. Similarly, the detection range of the northbound movement decreases from 88% between 50 ft and 100 ft to 14% between 100 ft and 150 ft from the stop bar. When the detection range extends from 150 ft to 200 ft from the stop bar, both the northbound and eastbound movements exhibit a detection rate of only 2%. In contrast, the southbound and westbound movements maintain good detection rates of 84% and 96%, respectively.

**Figure 9:**
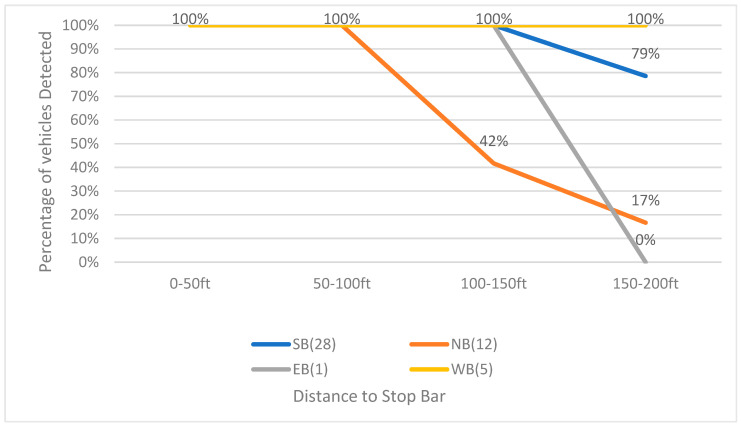
LiDAR-based vehicle detection rate for each bound (24 December 2020, 3:00 a.m.–3:30 a.m.).

As shown in Figure 9, LiDAR performed similarly during the night as the day: two bounds closer to the LiDAR installation location (northeast side of the intersection) show better performance, the westbound maintains a detection rate of 100% in four zones, and the southbound maintains detection rate of 100% in the first three zones (0 to 150 ft) and decreases to 79% in the fourth zone (150 to 200 ft). The performance of northbound and eastbound is slightly better than that in the daytime. The detection rate of eastbound is 100% in 0 to 150 ft to the stop bar, but it is 0% in the area of 150 to 200 ft to the stop bar. The northbound detection rate dropped from 100% to 42% at 100–150 ft to the stop bar and continued dropping to 17% at 150–200 ft to the stop bar.

**Figure 10:**
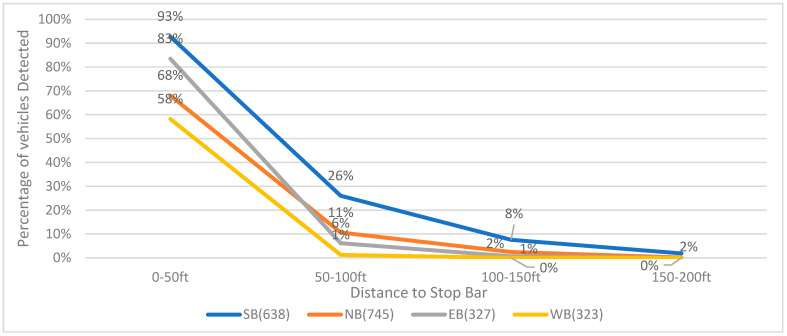
Vision-based vehicle detection rate for each bound (23 December 2020, 12:00 p.m.–12:30 p.m.).

Figure 10 shows the detection rate of cameras from 23 December 2020, 12:00 p.m. to 12:30 p.m. It can be observed that even within the area of 0 to 50 ft from the stop bar, there are instances where some vehicles cannot be detected by cameras. This suggests that a portion of vehicles can only be detected after they have entered the intersection. As the distance to the stop bar increases beyond 50 ft, the detection rates for all four movements sharply decline. Very few vehicles can be detected when they are over 100 ft away from the stop bar.

**Figure 11:**
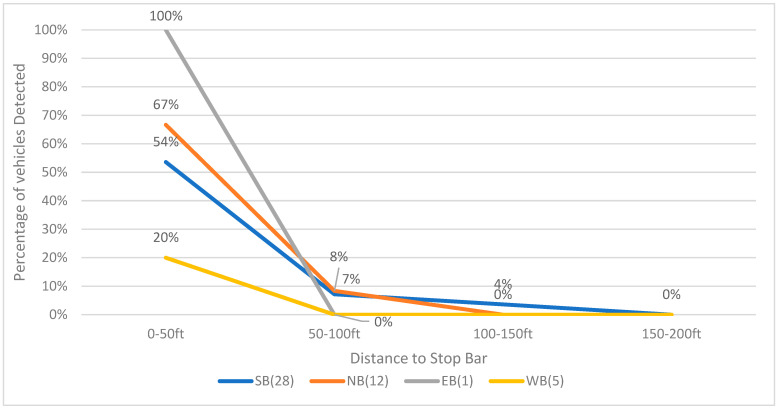
Vision-based vehicle detection rate for each bound (24 December 2020, 3:00 a.m.–3:30 a.m.).

The performance of the vision-based trajectory detection rate at night is worse than that in the daytime. As shown in Figure 11, for westbound, only 20% of the vehicles (1 vehicle out of 5) can be detected before reaching the stop bar. The detection rates of both eastbound and westbound are 0 when the detection range is 50 ft or longer to the stop bar, and that of northbound and eastbound is only 8% and 7%, respectively.

### 3.4. Pedestrian Detection

**Figure 12:**
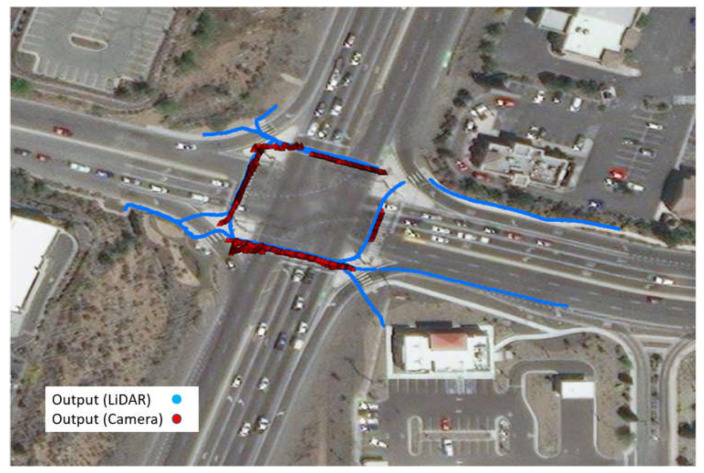
Pedestrian crossing trajectories captured by LiDAR (blue dots) and computer vision (Red triangles).

Pedestrian detection is crucial for traffic safety analysis. In this study, data from a 4 h daytime period and a 4 h nighttime period were selected to compare the performance of the two sensors in detecting pedestrians. Figure 12 illustrates the trajectories of pedestrian crossings from 23 December 2020, 12:00 p.m. to 4:00 p.m. By comparing the results with manually counted values, it is evident that both LiDAR and computer vision accurately captured all pedestrian activities. LiDAR was able to detect pedestrians before they entered the intersection, whereas the camera-based trajectories showed shorter lengths. Some pedestrians were only captured after they had already entered the crosswalk, as depicted in Figure 12.

During the nighttime period from 24 December 2020, 12:00 a.m. to 4:00 a.m., there were four pedestrian crossings recorded. Among them, all four pedestrians were successfully captured by the LiDAR sensor. However, none of these pedestrian crossings were captured by the camera sensor.

### 3.5. Speed 

**Figure 13:**
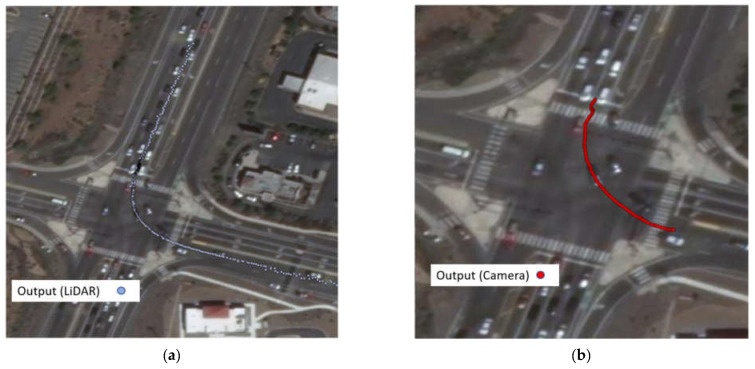
(**a**) Same vehicle trajectories captured by LiDAR; (**b**) same vehicle trajectories captured by computer vision.

**Figure 14:**
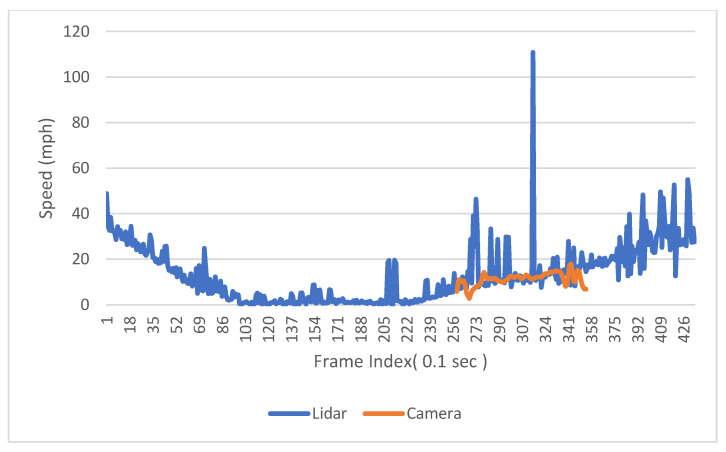
The speed of the sample vehicle calculated based on trajectory location.

**Figure 15:**
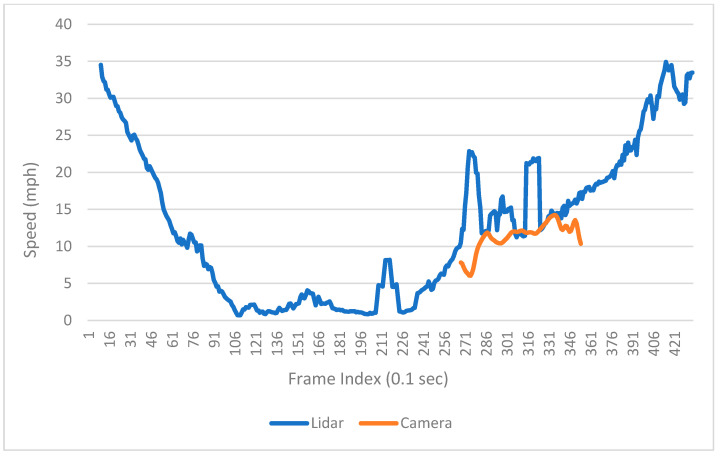
Smoothed speed of the sample vehicle.

The speed information can be calculated by calculating the distance difference over time difference. Figure 13 shows the trajectories of one southbound left-turn vehicle captured from both LiDAR and Video. Based on the trajectory data, the speed of the sample vehicle in each frame is calculated, which is shown in Figure 14. Since the offset of the location information can significantly affect the result of calculated speed, the speed is smoothed by the MA (Moving Average) method to get closer to the real value. The raw speed and smoothed speed information are shown in Figure 15.

From the raw data, it can be observed that the magnitudes of speeds calculated by the two sensors are similar. LiDAR exhibits greater fluctuation in speed, but it provides a longer trajectory length compared to computer vision. The trajectory captured by LiDAR encompasses the entire process of the vehicle, including deceleration towards the intersection, stopping at the intersection, and subsequent acceleration out of the intersection. This comprehensive trajectory information is crucial for intersection-level analysis.

**Figure 16:**
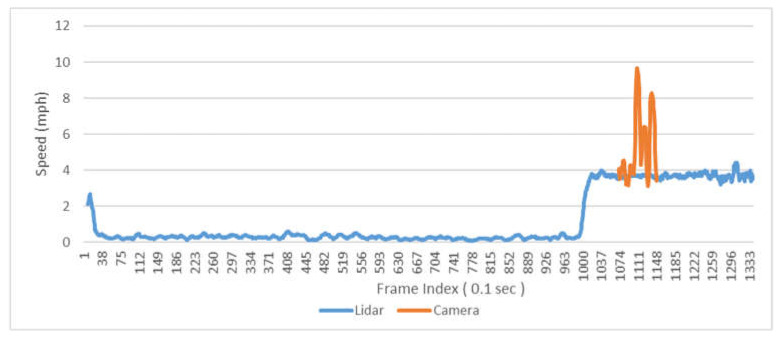
Sample pedestrian speed.

**Figure 17:**
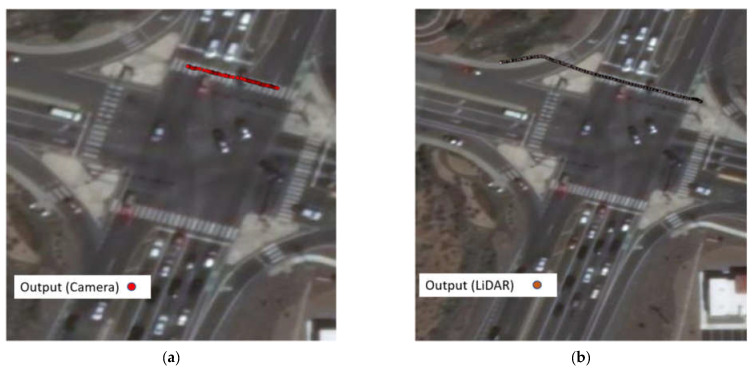
(**a**) Sample pedestrian trajectories captured by camera; (**b**) sample pedestrian trajectories captured by LiDAR.

For pedestrian detection, since the size of the pedestrian is much smaller than that of vehicles, the point reflected from pedestrians that LiDAR detects will also be significantly reduced, thereby reducing the potential distance offsets caused by the fluctuation of the reference point chosen on the object. This results in more accurate pedestrian speed detection by LiDAR compared to vehicle speed detection. As shown in Figure 16, LiDAR shows very good speed detection of pedestrians. On the contrary, the camera does not perform well in speed detection of pedestrians; the detected pedestrian speed is significantly higher than the normal walking speed of pedestrians (around 3–4 mph). Moreover, due to the short detection range of the camera, the camera can only capture the data of pedestrians walking on the crosswalk. As shown in Figure 17, the camera fails to capture pedestrians entering the island or accurately measure their waiting time before crossing.

Although the comparison of data indicates the superiority of current LiDAR-based trajectories, data quality is not the sole criterion when selecting a data source. Other factors, such as data storage, installation and maintenance accessibility, and cost, also need to be taken into account. Considering the data quality analysis and other relevant factors, Table 1 offers a general recommendation for researchers to consult when deciding which type to utilize. Cells marked with a single black dot signify that the respective data source exhibits good performance in that aspect; thus, the author suggests its use based on such considerations.

## 4. Conclusions

In conclusion, this study first outlines the significance of trajectory data within the transportation industry, then presents a comprehensive overview of the various sources and applications of trajectory data. This paper compares the leading LiDAR-based and computer vision-based multi-model all-traffic trajectory data generated on the market in terms of detection range, trajectory length, volume counting, and speed in different lighting conditions. 

The detection range of LiDAR and cameras was evaluated by dividing each movement into four detection zones, with each zone spanning a length of 50 ft. LiDAR exhibited a higher detection rate within the range of 0 to 50 ft from the stop bar compared to cameras. As the distance increased, the detection rates for northbound and eastbound movements significantly declined for both LiDAR and cameras. However, LiDAR maintained better overall detection rates, especially for southbound and westbound movements. During the nighttime, LiDAR maintained good detection rates for most movements, while cameras struggled to detect vehicles beyond 50 ft from the stop bar.

The comparison of volume counting performance reveals that the LiDAR system excels in maintaining consistent 96%+ accuracy regardless of lighting conditions, including both daylight hours and nighttime. It demonstrates reliable vehicle and pedestrian detection capabilities in various lighting conditions. In contrast, the vision-based system performs well during the daytime but encounters challenges in accurately detecting pedestrians at night. It failed to detect all four pedestrians during the nighttime period.

Both the LiDAR and camera systems demonstrate acceptable accuracy in measuring vehicle speeds. After applying smoothing techniques to the data, both systems provide reliable speed measurements. However, it is worth noting that the vision-based trajectory data for pedestrian speeds exhibit significant fluctuations, which may require further analysis and refinement.

To summarize, the LiDAR system offers advantages in terms of detection range and resilience to lighting conditions, making it a favorable choice for applications requiring precise and consistent trajectory data. The camera system can also provide valuable data, especially for vehicle speed measurements, with the understanding of its limitations in pedestrian detection. Researchers and practitioners should consider these factors when selecting the most appropriate sensor for their specific needs.

This paper’s analysis is limited to the data output of a single intersection with low pedestrian traffic. To further understand the comparison between LiDAR-based and vision-based multi-model all-traffic trajectory data, more comprehensive studies are necessary, including those involving urban intersections with high pedestrian traffic. The camera may prove to be more accurate and efficient in these situations due to its improved pedestrian recognition capabilities.

## Figures and Tables

**Table 1:** Summary of LiDAR-based and vision-based trajectory data collection and data quality.

Aspect	LiDAR-Based	Vision-Based	Comments
Hardware Cost		●	Cameras are currently much cheaper than LiDAR
Maintenance Cost		●	Video Camera is relatively easier to install and maintain than LiDAR; once a LiDAR is broken, it must be sent back to the manufacturer.
Software (data processing) Cost			The data processing costs for LiDAR and cameras are similar; both are expensive.
Data storage	●		Video needs much more storage space for the same time period than LiDAR data.
Detection Range	●		LiDAR shows a longer detection range.
Daytime Vehicle Volume	●	●	Both sensors show good daytime vehicle counting capability.
Nighttime Vehicle Volume	●		Cameras may miss some vehicles at night.
Daytime Pedestrian Volume	●	●	Both sensors show good daytime pedestrian counting performance.
Nighttime Pedestrian Volume	●		The camera barely recognizes pedestrians in poor-lighting conditions.
Vehicle Speed	●	●	Both sensors generate decent vehicle speed information.
Pedestrian Speed	●		LiDAR shows brilliant speed detection for relatively small objects, such as pedestrians/bicyclists.

## Data Availability

Not applicable.
